# Strategies for neural control of prosthetic limbs: from electrode interfacing to 3D printing

**DOI:** 10.3390/ma12121927

**Published:** 2019-06-14

**Authors:** Catherine G.Y. Ngan, Rob M.I. Kapsa, Peter F.M. Choong

**Affiliations:** 1 Department of Surgery, The University of Melbourne, St Vincent’s Hospital, Melbourne 3065, VIC, Australia; sarcoma@bigpond.net.au; 2 Biofab3D@ACMD, St Vincent’s Hospital Melbourne, Melbourne 3065, VIC, Australia; rmik@unimelb.edu.au; 3 ARC Centre of Excellence for Electromaterials Science, Intelligent Polymer Research Institute, Innovation Campus, University of Wollongong, Wollongong 2500, NSW, Australia; 4 Department of Medicine, The University of Melbourne, Melbourne 3065, VIC, Australia; 5 Department of Clinical Neurosciences, St Vincent’s Hospital, Melbourne 3065, VIC, Australia; 6 Department of Orthopaedics, St Vincent’s Hospital, Melbourne 3065, VIC, Australia

**Keywords:** neuroprosthetic interfacing, artificial limbs, myoelectric control, 3D printing, tissue engineering, bioprinting

## Abstract

Limb amputation is a major cause of disability in our community, for which motorised prosthetic devices offer a return to function and independence. With the commercialisation and increasing availability of advanced motorised prosthetic technologies, there is a consumer need and clinical drive for intuitive user control. In this context, rapid additive fabrication/prototyping capacities and biofabrication protocols embrace a highly-personalised medicine doctrine that marries specific patient biology and anatomy to high-end prosthetic design, manufacture and functionality. Commercially-available prosthetic models utilise surface electrodes that are limited by their disconnect between mind and device. As such, alternative strategies of mind–prosthetic interfacing have been explored to purposefully drive the prosthetic limb. This review investigates mind to machine interfacing strategies, with a focus on the biological challenges of long-term harnessing of the user’s cerebral commands to drive actuation/movement in electronic prostheses. It covers the limitations of skin, peripheral nerve and brain interfacing electrodes, and in particular the challenges of minimising the foreign-body response, as well as a new strategy of grafting muscle onto residual peripheral nerves. In conjunction, this review also investigates the applicability of additive tissue engineering at the nerve-electrode boundary, which has led to pioneering work in neural regeneration and bioelectrode development for applications at the neuroprosthetic interface.

## 1. Introduction

From Captain Hook to Luke Skywalker, both cinema and science have evolved in their respective visionary prescience of replacing a missing limb with a functional prosthetic device. Losing a limb can be a consequence of an unexpected traumatic event, such as in accidents or in combat [1,2]. At other times, it is a calculated decision of life over limb in the setting of tumour growth or other debilitating disease processes [3,4]. Facing amputation is a devastating experience with immediate and profound impact on the patient’s quality of life, as well as for their family, friends and carers [3]. For the surgeon, these operations are a poignant reminder of our limitations in medicine despite all the advances of modern technology. It does, however, serve as powerful inspiration to turn the science-fiction of dextrous, body-integrated robotic limbs into a reality.

Long-standing ‘peg-leg’ and ‘hand-hook’ prosthetics fulfil simple mechanical requirements and can adequately restore gross motor functions such as standing and walking. These prostheses are immediately amenable to modern additive fabrication technologies, which is evident in the recent establishment of numerous manufacturing ventures to rapidly 3D-print bespoke, cost-effective functional prosthetics for amputees [4,5]. These activities will in future inevitably traverse a marketplace that encompasses major as well as a smaller commercial entities to provide prosthetics of varying complexity to furnish simple mechano-structural functionality in cases of lost limbs.

In a similar light, advanced prosthetic technologies with multiple motorised joints have long been developed and, in fact, are commercially available. Again, advancements in additive fabrication technologies will inevitably lead to high-throughput fabrication of limb prostheses on a personalised basis. Unfortunately, their clinical application has been limited due to the fact that these devices must presently largely be driven without true intuitive motor intent. Examples include prosthetic limbs that are activated by surface electrodes on remaining muscle groups, or even manual changes of motion through buttons and smartphone apps [6]. This results in a disconnect between what the brain wants the robotic limb to do, and how the device is programmed to respond. Thus the critical obstacle for the adoption of this technology is the interface between the mind and the prosthetic device.

This literature review thus investigates current neuroprosthetic interfacing technologies, with a focus on long-term integration with endogenous host biology, harnessing the user’s cerebral commands and how additive biofabrication technologies may ultimately enable achievements of these functions. It investigates the limitations of skin, peripheral nerve and brain interfacing electrodes, and in particular the challenges of minimising the foreign-body response, as well as a new innovative strategy of grafting muscle onto residual peripheral nerves. It also introduces the concept of integrated bio-prosthetic engineering at the nerve-electrode boundary, which has led to pioneering work in neural regeneration and bioelectrode development for applications at the neuroprosthetic interface.

## 2. The Motorised Prosthetic Limb

Prosthetic devices play an important role in rehabilitation after amputation [7]. At a minimum, they can serve as cosmetic restoration, which can provide psychological benefit and have simple passive or opposing functions [8]. More advanced prosthetics offer some functional restoration, enabling activities of daily living such as grooming and dressing, and minimising injury on the contralateral side from compensation and overuse [9,10]. For the return of basic function, body-powered prosthetics utilise movement of a proximal joint to operate a terminal device [11]. With this method, the patient uses movement in the remaining limb to trigger a physiologically unrelated action in the prosthesis e.g. shoulder flexion and extension may trigger a prosthetic pincer to open and close. Although simple and effective, this system of pulleys and cables typically allows only one joint to be operated at a time [11]. More sophisticated function requires simultaneous articulated movements, in particular for the human hand [12]. As such, motorised prosthetic limbs have been developed to offer greater mobility, dexterity and motor control than purely structural, non-motorised prosthetics.

**Figure 1:**
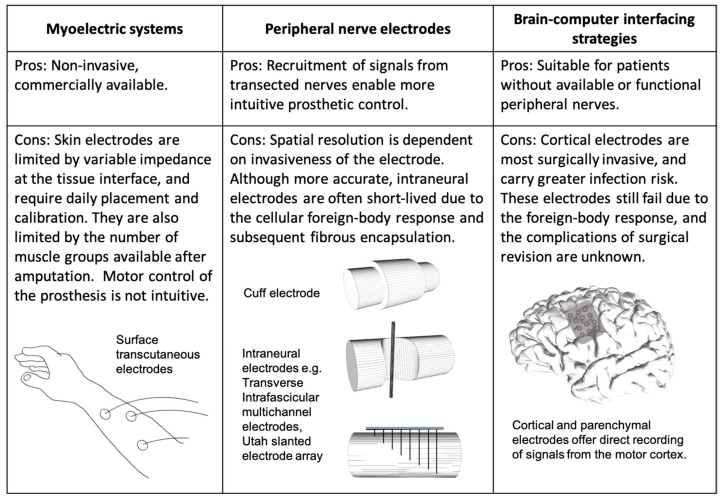
Different methods of achieving mind-control of motorised prosthetic limbs.

Robotic arms with multiple degrees of freedom (DOF) and a variety of terminal grippers have long been used in the manufacturing industry [13]. Similarly, advanced motorised prosthetic limbs have also been designed with articulated humanoid hands and accompanying grip sequences [14,15]. These are readily prototyped and produced with existing 3D printing technology, requiring low levels of regulatory approval to translate [16]. Despite these mechatronic and additive biofabrication advances, the real challenge for achieving functionality of a human prosthetic limb is its integration with functional biology. Ideally, the prosthesis should be driven by the user’s motor intent and thus respond to purposeful cerebral commands. Efforts to achieve biological control of such bionic systems have led to the development of different neuroprosthetic interfacing strategies. These interfacing electrodes can be broadly categorised by site: on remaining muscle groups, in the peripheral nervous system (PNS), and in the central nervous system (CNS) (Figure 1).

### 2.1. The Myoelectric Prosthesis

The most common approach to neuroprosthetic interfacing is to utilise the innervation of remaining muscle groups after an amputation [11]. Surface electrodes applied to the skin detect activation of muscle groups below to trigger the robotic limb. Their main advantage is adaptability and non-invasiveness, and as such surface electrodes are used in commercially available devices such as the i-Limb systems (Touch Bionics), the Michelangelo Hand (Ottobock US) and the SmartHand (The Biorobotics Institute) [6,17,18,19]. These examples mostly pertain to the upper limb, given the unique challenges that come with restoring dexterity and function of the hand. A significant barrier to the successful implementation of these prostheses is the difficulty of faithful transmission from muscle to electrode. Limiting factors include variable impedance at the skin–electrode interface, the need for daily placement and calibration, and the limited number of muscle groups available after amputation [16,17]. The latter problem is worsened by higher amputations, for which the robotic limb requires more signalling points than are available.

In an effort to retain signalling points, transected nerves after limb amputation can be transferred to remaining muscle groups in a surgical procedure known as Targeted Muscle Reinnervation (TMR). In a technique first described in 2004 by Kuiken and Dumanian, residual nerves from the limb can be transferred to denervated sections of nearby muscle [20]. This method demonstrates the remarkable capacity of muscle to accept innervation from different nerves and has the added benefit of amplifying neural signals and mitigating the formation of painful neuromas [21]. Most importantly, the signals from the transected nerve are preserved in several new myoelectric control sites [21]. Another fortuitous finding from these surgeries is that the transferred nerve can also provide sensation to the overlying skin, offering potential sites for sensory feedback from the prosthesis [20]. While TMR is a promising technique for the preservation of neural signals, it faces the same issues as traditional myoelectric systems, which include the skin–electrode connection, wearing multiple electrodes, and still a limited overall number of signalling points for triggering the robotic limb. Furthermore, these strategies are restricted by size and suitability of surrounding muscle, demand substantial recovery time and are not truly representative of the user’s intent [22]. 

An alternative to surface electrodes for the detection of muscle activity is the Implantable Myoelectric Sensor (IMES) System [23]. IMES electrodes are small cylinders (16 mm long, 2.5 mm in diameter) that wirelessly transmit myoelectric activity after insertion in the muscle belly. The IMES system uses six implanted electrodes to allow for simultaneous control of three DOFs, and can detect activation of deeper muscles not previously recruited with surface recordings. This system also promises more comfort for the wearer as it does not depend on a tight electrode-skin interface, and so the prosthesis can be optimised for comfort. The issues with this system are again the same as with all current myoelectric prosthetic devices; there are limited muscle groups available after amputation, and the system is not intuitive if the neural signals from the transected nerve are not harnessed. 

### 2.2. Peripheral Nerve Electrodes

Another strategy to achieve prosthetic control is to record directly from peripheral nerves. Electrodes can be applied either extraneurally or intraneurally to detect signals that reflect the user’s motor intention. Extraneural electrodes, such as the cuff electrode or Flat Interface Nerve Electrode (FINE), wrap around the entire nerve bundle and record changes in electrical potential on the perimeter of the nerve. As such, its spatial resolution is limited due to its superficial position. Cuff electrodes have been shown to withstand chronic implantation in human peripheral nerve studies, and have applications for long-term neural stimulation to treat conditions such as chronic pain or bladder dysfunction [24,25,26]. However, its poor selectivity of recordings from individual fascicles makes it less suitable for interpreting the variety of neural signals required to trigger a robotic limb.

Higher spatial resolution can be achieved through penetrating intraneural electrode systems. Longitudinal Intrafascicular Electrodes (LIFE), Transverse Intrafascicular Multichannel Electrodes (TIME) and the Utah Slanted Electrode Array (USEA) are all examples of electrode systems that pierce the epineurium and record from a number of fascicles within the nerve bundle [27,28,29]. LIFE and TIME are essentially single wires with several recording points, which are inserted longitudinally or transversally to address multiple fascicles. This approach, particularly longitudinal insertion, minimises surgical damage, and these systems have been successfully implanted for up to three months in animal studies [30]. There is little data on the recording capabilities of these electrodes, but stimulation studies show promise for recruiting multiple muscle groups [31]. The USEA is the most invasive example, consisting of an array of electrodes from short to long inserted into the nerve. These electrodes have the highest spatial resolution, with the most fascicles within recording distance of an electrode. Its high recording fidelity was demonstrated in two human subjects, who after implantation of the USEA into their remaining upper limb nerves were able to move individual fingers on a virtual robotic hand [32]. Its main issue, however, is the damage caused to the nerve after chronic implantation, due to its invasiveness and issues with micromotion across the array [33,34]. 

The last peripheral nerve interfacing strategy is the regenerative electrode. These systems can be described as sieves or hollow electrode array systems, which are placed between two ends of a severed nerve [35]. The aim is for the nerve to regenerate through the porous electrode, thus optimising its resolution. The requirement for the nerve to be severed, followed by a highly variable process of neural regeneration, makes this technology the most invasive of the peripheral nerve electrodes. However, early animal experiments have demonstrated some neural regeneration over three to four months after implantation, and that these electrodes can both record and stimulate [36,37].

### 2.3. Brain–Computer Interfaces

Brain–computer interfaces (BCI) record signals from the CNS to drive the robotic limb. The invasive nature of these techniques mean that it is mostly limited to subjects with severe disease, such as paraplegia or quadriplegia, and that they are perhaps less applicable to amputees who are otherwise systemically well. Nonetheless, it is an important interfacing strategy to consider in the scope of neuroprosthetic technology. In conditions where peripheral nerves are not available due to spinal or brain injury, or severe neuromuscular degeneration, motor intent can be recorded from the brain itself. Recording electrodes can be categorised by invasiveness, ranging from surface or scalp recordings (electroencephalography—EEG), cortical recordings (electrocorticography—ECoG), or within the parenchyma of the brain itself (local field potentials and single-neuron action potential recordings).

EEG is a non-invasive approach that utilises surface electrodes to detect brain activity through the scalp. Although it is the safest method of recording brain activity, the centimetre’s distance of the scalp from the brain and the poor transmission through cerebrospinal fluid, skull and skin greatly hinders the spatial resolution [38]. EEGs have nonetheless been a useful tool for providing a channel of communication through the control of computer cursors and spelling programs for patients with severe paralysis, and were a starting point for the interpretation of cortical activity to drive prosthetic devices [39,40,41]. Higher resolution recordings can be obtained with ECoG BCI systems, which require surgical implantation of an electrode array on the surface of the brain. ECoG systems have been used for decades to intraoperatively identify epileptogenic areas for surgical removal in patients with intractable seizures [42,43]. More recently, this technology has been used to record motor cortex activity to control upper limb prosthetics, even achieving individual finger control [44,45,46].

The highest resolution is achieved with the most invasive approach, which is to implant penetrating electrodes into the motor cortex. These electrode arrays are able to record local field potential activity or even individual neurons within the brain. Although the high spatial resolution reflects more intuitive and accurate motor function, their significant limitation is in maintaining stable long-term recordings due to the inflammatory reaction and fibrotic capsule formation around the implant [47]. Despite this, invasive electrodes have been used to direct motion in some of the most advanced prosthetic limbs, including the DEKA Arm System (Mobius Bionics). Ninety-six-channel microelectrode arrays were implanted into the motor cortex hand area of two study participants with longstanding quadriplegia, both of whom were able to operate the DEKA arm to touch and grasp objects [48]. This breakthrough experiment offered a glimpse into the possibilities of BCI interfacing in a prosthetic arm that boasts ten powered DOFs [14]. However, this level of control has not yet been achieved with BCI recordings alone, and indeed the DEKA arm has been made commercially available as a myoelectric prosthesis coupled with foot controllers [49]. 

The main resistance to widely applying BCI systems is the obvious surgical and infection risk that comes with craniotomies for electrode implantation in the CNS. To counter this, a recent innovation in neural interfacing technology is the development of an electrode array delivered as an endovascular stent [50]. Through standard angiography techniques, this device can be delivered and positioned within the cerebral vasculature to record signals at a resolution similar to ECoG recordings. This minimally invasive technique has been trialled in the sheep model, where electrodes were delivered in the cerebral venous system and implanted for up to 190 days [51]. Its potential in BCI systems, seizure prediction in epilepsy and deep brain stimulation therapies remain to be explored.

## 3. The Challenges of Tissue-Electronic Interfacing

Whether an electrode is positioned in muscle, nerve or brain, its interaction with the surrounding environment will contribute significantly to its effectiveness and longevity. Minimising the physiological foreign-body response (FBR) is key to designing any biomedical implant [52]. Outside of the CNS, FBR begins with the immediate deposition of host proteins on the surface, such as albumin, fibrinogen, complement, and fibronectin, to initiate the acute inflammatory cellular reaction [53]. The acute phase is characterised by the presence of neutrophils and histamine release from mast cells. Together, these cells and their bioactive agents mediate the initial reaction to an implant, further recruiting monocytes and macrophages to the site [54]. The deposition of protein provides a means of attachment for macrophages, which can fuse into foreign-body giant cells (FBGC) for the phagocytosis of larger particles [55]. Biomedical implant devices are typically well beyond the phagocytic capacity of cells, and in response, these cells release reactive oxygen intermediates, enzymes and acid into their environment [56]. Eventually, the over-activation of inflammatory cells leads to the excessive deposition of collagen from fibroblasts in a process known as fibrous encapsulation, thus walling off the device and often causing device failure [57].

Of relevance to BCI systems, the foreign-body response within the CNS can be similarly destructive, with some site-related differences. Although sometimes labelled an ‘immune privileged’ site, the CNS in fact has an extensive innate immune response, mediated by microglia, oligodendrocytes and astrocytes, that is similarly capable of mounting a FBR [58,59,60]. In addition, the trauma of implantation disrupts the cerebral vasculature, breaking what is known as the blood–brain barrier (BBB), which introduces blood-borne immune cells to the CNS [61]. While the timeframe of the FBR response in the CNS may be longer and the overall inflammation somewhat reduced, the inevitable neuronal cell death and potential for infection are serious risks that must be considered [62,63].

Design considerations to minimise the FBR include matching the material to the surrounding mechanical stiffness of the tissue, accommodating for any contractile dynamics, and conforming to the topography of the tissue to maximise contact area [64]. For long-term neural interfacing, the main challenge is to design implants that mimic the very soft modulus of neural tissue [65]. Traditional platinum or gold electrode probes are unsuitable for this environment, as they tend to provoke a chronic inflammatory response with cell death of target neurons [62,63]. As such, long-term invasive recording systems are difficult to sustain in this delicate neuronal microenvironment, which is sensitive to disturbance and quick to respond with cell death and scarring. Minimising invasiveness, as mentioned with the LIFE and TIME electrode wires, can prolong its lifespan but at the cost of recording resolution. More general strategies can be adopted, which include coating electrodes with anti-inflammatories, surface modification with hydrophilic polymers, or using anti-adhesive coatings [66,67,68]. Despite these advances, long-term neural interfacing is a bioengineering conundrum constantly challenged by delicate neural biology.

As an alternative, using skeletal muscle as a surrogate for neural interfacing is an attractive option, although it does present its own unique challenges. Muscle is a much larger and tougher structure than nerve, with greater regenerative potential, more metabolic reserve and stiffer mechanical properties [69,70,71,72]. The amplitude of EMG signals is far greater than neural signals and thus easier to record [73]. Its main physiological limitation is its contractile nature, which can shorten the tissue by up to 20% of its original length [74]. As such, electrodes that are highly flexible and elastic are required. Conducting polymers that can withstand high strains over many cycles have been investigated, including polypyrrole, polyaniline and poly(3,4-ethylenedioxythiophene) (PEDOT) [75,76,77]. Alternatively, non-conductive flexible materials such as polydimethyl-siloxane (PDMS) or styrene-butadiene-styrene (SBS) can be combined with conductive nanoparticles [78,79]. Buckling the conductive material on pre-stretched substrates can prevent cracking of the electrode when implanted in contractile environments, and placement of electrodes in a more mechanically neutral plane can also minimise the stress on the material [75]. Of note, interfacing with contractile muscle has been achieved in clinical practice for decades in the cardiac pacemaker, where its lead wires are typically implanted into the endocardial wall of the heart and can last the lifetime of the patient [80]. Given the much lower degree of tissue injury and surgical risk, electrode-interfacing with muscle is biologically more achievable. However, without harnessing the signals from residual nerves to the missing limb, this strategy will never lead to intuitive prosthetic motor control.

## 4. Grafting Skeletal Muscle onto Residual Nerves

A solution that marries the biological advantages of muscle with the preservation of neural signals is the development of a muscle-based Regenerative Peripheral Nerve Interface (RPNI). The RPNI is an autologous muscle graft neurotised by the free end of the residual nerve [81]. Rat and macaque studies have demonstrated that peripheral nerves can be dissected down to its fascicles for attachment to individual RPNIs which become innervated over three to four months [82,83]. In contrast to TMR, this technique can be applied to any level of amputation and is less constrained by patient anatomy. The intraneural dissection also allows for more specific functionality of the muscle recording, which is key to intuitive prosthetic control. Most recently, RPNIs with intramuscular recording electrodes were implanted in macaques for up to twenty months. This proof-of-concept study demonstrated the ability of muscle grafts to be successfully innervated, tolerate chronic electrode implantation, and record signals that could be decoded to represent individual finger movement [84].

RPNIs consist of small muscle grafts (~1 cm × 3 cm) that are transposed to residual nerves without a dedicated blood supply. This has been reported as being adequate for tissue survival, with normal muscle histology present after four months of implantation [83]. The process of innervation of these grafts is currently in the order of months in small animal models. The authors also noted that some RPNIs reintegrated into surrounding muscle, making it difficult to isolate EMG signals after twenty months of implantation [83]. Aside from these limitations, the RPNI is a creative method of side-stepping the challenges of neural biology, and early functional animal studies show great promise for achieving sophisticated neuroprosthetic control.

## 5. Tissue Engineering in the Neuroprosthetic Interface

The drawback to the RPNI is that it relies on grafted muscle, which requires the harvest of functional, healthy tissue from a patient who has already suffered a significant loss. Moreover, it does not specifically promote the process of innervation, which is the critical purpose for using such a graft. An alternative may be to tissue engineer the necessary autologous nerve or muscle through the combination of progenitor cells, scaffold material and neurotrophic biochemical cues.

Methods of bridging the biological mismatch of tissue and electrode have been explored with hydrogel polymers, which have the advantage of being amenable to mechanical and biochemical modification. Blending hydrogels with conductive polymers, such as polypyrrole or PEDOT, have been explored as a method of mediating the modulus mismatch and providing desirable electrical characteristics. While attractive in theory, these hybrid materials are limited in their ability to support neural regeneration and integration and have shown low functionality in short-term in vivo studies [85,86,87]. To improve neuronal survival at the electrode surface, a progression from this idea is to combine hydrogels with neural progenitors and biofactors to create a ‘living electrode’. This approach adopts the principles of tissue engineering, with the goal of growing an electrode-integrated layer of fabricated neural tissue to regenerate with transected nerves. Frontier work in this field has led to preliminary studies demonstrating the concept of neural networks encapsulated in degradable hydrogels on electrodes [88]. Their functionality is yet to be explored.

Similarly, early efforts to use tissue-engineered muscle for grafting onto transected nerves have shown promise for the recording of neural signals. Silicon, acellular extracellular matrix (ECM) and ECM with PEDOT were tested as suitable scaffolds for myotube grafting onto a transected nerve in vivo [89]. These artificial muscle grafts were attached to nerves in the same manner as RPNI constructs, and after an average implantation time of 93 days, myoelectric activity was recorded in response to neural stimulation. Although the biology of this construct was rudimentary and the inclusion of PEDOT did not enhance the muscle or innervation, it was able to demonstrate the concept of growing autologous muscle for grafting onto transected nerves, as opposed to harvesting tissue from elsewhere in the body.

## 6. Bioprinting and 3D Printing

The process of tissue engineering opens up opportunities to integrate electrode systems into living structures as part of the fabrication process, ultimately creating bioelectrode devices personalised to the individual’s anatomy and biology. While there are a variety of techniques to engineer nerve and muscle, bioprinting is a particularly promising approach for the fabrication of 3D tissues for integration into bioelectronic systems. It involves the deposition of living cells in additive layers, using a computer-controlled system to precisely create a pre-planned 3D structure [90]. Current 3D bioprinting strategies range from laser bioprinting, piezoelectric bioprinting and extrusion bioprinting [91,92]. For creating structures that can be surgically grafted, and in particular skeletal muscle, extrusion bioprinting has the advantage of being the only system to deliver high cell density constructs in a scalable manner.

Extrusion bioprinting uses a ‘bioink’ of cells encapsulated in a hydrogel, which is deposited as layered filaments to create custom-shaped constructs. The extrusion system typically uses mechanical or pneumatic pressure to control the flow of bioink through a nozzle that can print down to a resolution of 100 µm [90]. It enables rapid fabrication of cell-laden hydrogel filaments, and furthermore allows for precise patterning and construction of highly porous networks. This overcomes the problem of diffusion distance and offers more opportunity for the infiltration of vasculature and nerves. Although there are few examples of functional bioprinted muscle or nerve, early results highlight the engineering versatility of this technique, although acknowledging the requirement for further refinement of the scaffold material and cell culture conditions [93]. In combination with the aforementioned electrode technology, the interwoven fabrication of engineered tissue and interfacing electrodes points to exciting future developments for neuroprosthetic interfacing.

The process of additive manufacturing also extends to the fabrication of the electrode devices themselves. 3D printing has facilitated rapid production of bespoke laboratory and medical equipment, with new opportunities for tissue engineering applications [94,95,96]. It offers creative solutions for cell cultures systems that might include microfluidics, perfusion technology, and integration of electrodes into 3D printed devices [97,98,99]. Of relevance to bioelectrode development and tissue-electrode interfacing, recent studies have demonstrated the feasibility of 3D printing graphene-based electrodes, carbon-fibre microelectrodes and stainless steel electrodes [100,101,102]. Another approach is to 3D print the device to house the electrode wires [99]. Recognition of imminent innovations in design and bioengineering led the authors of this paper to publish on the biocompatibility of commonly used photopolymer 3D-printing inks, and methods of optimizing these materials for cell culture [103]. Such advances in electrode and device fabrication techniques easily translate to enhancement of tissue engineering strategies, with obvious creative scope for the simultaneous fabrication of integrated electrode and cellular structures as a single bioelectrode device.

## 7. Conclusions

With the commercialisation and increasing availability of advanced motorised prosthetic technologies, there is a consumer need and clinical drive for intuitive user control. Current models that utilise surface electrodes are limited by their disconnect between mind and prosthetic device. Direct neural interfacing, whether it is in the central or peripheral nervous system, has shown that intuitive control can indeed be achieved. However, the longevity of such systems is challenged by the foreign-body response, which leads to fibrous encapsulation of the electrodes, neuronal cell death and failure of the device. To overcome this problem, skeletal muscle has been explored as an alternative recording source. Neurotised muscle grafts have been demonstrated to amplify neural signals and have shown potential for side-stepping the challenges of long-term neural interfacing. Lastly, the scope for direct applications of additive biofabrication technologies, such as 3D rapid-prototyping and bioprinting, in the manufacture of prosthetic limbs are ever more obvious. Advances in tissue engineering have led to neural and muscle engineering specifically at the neuroprosthetic interface, which open up creative possibilities for the integration of innervated tissue with electrodes and the opportunity to tailor these interfacing systems to the user’s own biology and anatomy. In combination with advances in biomaterial development and signal processing algorithms, this will likely lead the way to long-term intuitive motor control of prosthetic limbs.
